# The Genetic Architecture of Bovine Telomere Length in Early Life and Association With Animal Fitness

**DOI:** 10.3389/fgene.2019.01048

**Published:** 2019-10-25

**Authors:** Joanna J. Ilska-Warner, Androniki Psifidi, Luise A. Seeker, Rachael V. Wilbourn, Sarah L. Underwood, Jennifer Fairlie, Bruce Whitelaw, Daniel H. Nussey, Mike P. Coffey, Georgios Banos

**Affiliations:** ^1^Animal and Veterinary Sciences, Scotland’s Rural College, Edinburgh, United Kingdom; ^2^The Roslin Institute and Royal (Dick) School of Veterinary Studies, University of Edinburgh, Edinburgh, United Kingdom; ^3^Royal Veterinary College, University of London, London, United Kingdom; ^4^MRC Centre for Regenerative Medicine, University of Edinburgh, Edinburgh, United Kingdom; ^5^Institute of Evolutionary Biology, School of Biological Sciences, University of Edinburgh, Edinburgh, United Kingdom

**Keywords:** telomere length, bovine, genetic parameters, GWAS, animal fitness, longevity

## Abstract

Health and survival are key goals for selective breeding in farm animals. Progress, however, is often limited by the low heritability of these animal fitness traits in addition to measurement difficulties. In this respect, relevant early-life biomarkers may be useful for breeding purposes. Telomere length (TL), measured in leukocytes, is a good candidate biomarker since TL has been associated with health, ageing, and stress in humans and other species. However, telomere studies are very limited in farm animals. Here, we examined the genetic background, genomic architecture, and factors affecting bovine TL measurements in early life, and the association of the latter with animal fitness traits expressed later in life associated with survival, longevity, health, and reproduction. We studied two TL measurements, one at birth (TLB) and another during the first lactation (TLFL) of a cow. We performed a genome-wide association study of dairy cattle TL, the first in a non-human species, and found that TLB and TLFL are complex, polygenic, moderately heritable, and highly correlated traits. However, genomic associations with distinct chromosomal regions were identified for the two traits suggesting that their genomic architecture is not identical. This is reflected in changes in TL throughout an individual’s life. TLB had a significant association with survival, length of productive life and future health status of the animal, and could be potentially used as an early-life biomarker for disease predisposition and longevity in dairy cattle.

## Introduction

Telomeres are evolutionarily conserved, tandemly repeated DNA sequences, which protect the ends of chromosomes during cell division (Blackburn, 1991). Due to the “end replication problem,” telomeric sequences are truncated during each mitotic cycle; when a critical length is reached, their protective function is lost resulting in chromosomal instability, and the cell enters a stage of replicative senescence and apoptosis, which may lead to progressive tissue atrophy (Counter et al., 1992; Campisi, 1997; Sahin et al., 2011).

Results from previous *in vitro* studies at cellular level and on model organisms have led to a hypothesis that telomere length (TL) could be used as a predictor of the individual organism’s lifespan. Indeed, this hypothesis has found support in studies on wild birds (Haussmann et al., 2005; Bize et al., 2009; Heidinger et al., 2012; Barrett et al., 2013), humans (Cawthon et al., 2003; Bakaysa et al., 2007), and other organisms (Brown et al., 2012; Olsen et al., 2014), which have collectively shown that shorter TL may be associated with reduced lifespan. Further studies have reported associations between TL and age-related disorders in humans, such as post-stroke dementia and a decline in cognitive ability (Martin-Ruiz et al., 2006), cardio-vascular disease (Cawthon et al., 2003; Brouilette et al., 2007), and certain kinds of cancer (Shammas, 2011).

As interest in telomeres in human medicine grew, TL was found to be associated with a multitude of conditions, not necessarily limited to those related with age. For example, short TL was found to be over-represented among patients with immune-compromised conditions such as AIDS (Auld et al., 2016). Short TL was also shown to be associated with fertility-related conditions such as estradiol levels in women with benign endometriosis (Hapangama et al., 2008) and occult ovarian insufficiency (Butts et al., 2009), or idiopathic infertility in men (Thilagavathi et al., 2013). Even in seemingly healthy individuals, TL has been used as an indicator of stress levels—for example, women exposed to prolonged and high levels of stress were found to have similar TL as women a decade older (Epel et al., 2004).

In non-human species, telomere studies are less common, with the majority concentrating on the use of TL as an indicator of life-history traits in wild populations (Asghar et al., 2014; Olsen et al., 2014; Angelier et al., 2015; Becker et al., 2015; Fairlie et al., 2015; Bauch et al., 2016) or as a predictor of the “genetic age” in cloned animals (Lanza et al., 2000; Tian et al., 2000; Jiang et al., 2004; Jeon et al., 2005).

Farm animals rarely reach their biological limits of lifespan and, therefore, their TL did not originally receive much research interest. However, as results from studies of TL in humans expand our knowledge on telomeres and their intricate connection with health, ageing and stress, telomeres are coming to the forefront as potential biomarkers for longevity (Brown et al., 2012), welfare (Bateson, 2016; Beloor et al., 2010), and metabolic stress (Laubenthal et al., 2016) in farm animal (livestock) species.

Health and survival are now key goal traits of selective breeding in farm animals, as illustrated by an increasing emphasis being placed on fitness in relevant genetic improvement programs (Agriculture and Horticulture Development Board, 2018). Progress, however, is often limited by the low heritability of fitness traits (Pritchard et al., 2013) combined with measurement difficulties. In dairy cattle, a considerable proportion of cows are involuntarily removed (culled) from the herds before reaching their full production potential (Brickell and Wathes, 2011) because of poor health and/or fertility, thus causing severe economic losses to the sector through high costs of raising a heifer (young cow) not being met by the expected returns from milk production of the adult cow. Therefore, a trait measured early in life, which could predict the future fitness of a cow, either as a biomarker or as a genetically correlated trait, would vastly improve the returns in dairy industry, while also addressing animal longevity and welfare. TL may be a likely candidate for such a trait. In two previous studies examining the phenotypic association between TL and dairy cow survival, significant and positive results were found (Brown et al., 2012; Seeker et al., 2018a). However, the genetic correlation between TL and fitness traits has not been studied before, even though there is now a considerable body of research demonstrating the presence of genetic variation in TL.

Heritability estimates of TL vary between studies and species. For example, heritability of TL in humans is estimated between 0.3 and 0.8 (Slagboom et al., 1994; Vasa-Nicotera et al., 2005; Andrew et al., 2006; Liinamo et al., 2007; Broer et al., 2013; Eisenberg, 2014; Faul et al., 2016; Sillanpää et al., 2016). Estimates in other species also show high variability, ranging from 0.2 in king penguins (Reichert et al., 2015) to 0.3–0.4 in dairy cows (Seeker et al., 2018b) to 0.77 in kakapos (Horn et al., 2011). While studies in various species consistently demonstrate a significant genetic component in TL variation, the exact inheritance patterns are still under discussion. Initial studies indicated possible X-linked inheritance (Nawrot et al., 2004), soon followed by the opposite findings supporting paternal inheritance (Nordfjäll et al., 2005; Njajou et al., 2007) or results indicating equal contributions from both parents (Atema et al., 2015). The paternal inheritance theory seemed to gain more support in the human studies (Nordfjäll et al., 2005; Njajou et al., 2007; Nordfjäll et al., 2009); however, epigenetic effects of the father’s age might have largely driven this theory (Bauch et al., 2019; Eisenberg, 2019). Furthermore, genome-wide association studies (GWAS) in humans identified several single nucleotide polymorphisms (SNPs) and genes associated with TL (Prescott et al., 2011; Codd et al., 2013) attesting to a possibly complex polygenic inheritance pattern for the trait.

The objectives of the present study were to i) examine the genetic architecture of early-life leukocyte TL measurements in dairy cattle using pedigree and genomic data and ii) determine the association of early-life bovine TL with future animal fitness traits, linked with health, reproduction, survival and longevity. We used the largest population of farm animals with telomere measurements published to date and conducted variance component analyses to estimate genetic parameters, and GWAS to identify genomic regions and candidate genes controlling TL. We also investigated the predictive capacity of TL with regards to animal health, reproduction, survival and longevity using mixed linear models.

## Materials and Methods

### Ethics Statement

Blood sample collection was conducted in accordance with UK Home Office regulations (PPL No: 60/4278 dairy systems, environment, and nutrition), and procedures were approved by the Scotland’s Rural College (SRUC) Animal Experimentation Committee. The other data used in the analyses were obtained from a database of records collected routinely at the experimental farm.

### Study Population

The population used in the present study consisted of female Holstein–Friesian cattle raised at the SRUC Dairy Research Centre at Crichton Royal Farm in Scotland, UK. The herd was established more than three decades ago to study the effects of long-term selection and the interactions between animal genotype and diet (Pryce et al., 1999). The cows are maintained in a two-by-two experimental design, with two genetic groups (selected group for increased milk protein and fat yield *vs*. control group maintained at the UK national average level) and two feeding groups (low forage diet based mainly on concentrated feed with cows housed throughout the year *vs*. high forage diet with cows turned out to pastures during summer). All animals are closely monitored and recorded for a range of traits and events (milk production, fertility, health, feed intake, calving data, veterinary treatments etc.). Since 2004, a routine blood sampling protocol for experimental animals has been established, with the first sample being collected within the first 2 weeks from birth and repeat sampling carried out annually.

The dataset used for the present study included 2,202 blood samples collected from 702 animals, born between 2008 and 2015. Animals were approximately equally split between genetic and feeding groups. A complete pedigree of 11,598 animals was extracted from the farm database.

### Measurement of Telomere Length

DNA was extracted from frozen whole blood samples using the DNeasy Blood & Tissue spin column protocol (QIAGEN) and had to pass the following quality control steps to be considered for TL measurement with qPCR: DNA yield, measured on a NanoDrop ND-1000 Spectrophotometer (Thermo Scientific), had to be greater than 20 ng/μl, and DNA purity, expressed as ratios of absorbance at 260nm/280nm and 260nm/230nm, had to be greater than 1.7 and 1.8, respectively. Furthermore, each sample had to pass a DNA integrity gel test described in Seeker et al. (2016).

The amount of telomeric DNA was measured in relation to the amount of the reference gene beta-2-microglobulin (B2M) that is constant in copy number and has been used before in telomere studies on ruminant species such as the Soay sheep (Fairlie et al., 2015; Watson et al., 2017), roe deer (Wilbourn et al., 2017), dairy cattle (Seeker et al., 2018a). The qPCR reactions for the telomere and reference gene amplification were performed on the same qPCR plate but in different wells (Monoplex qPCR). On each qPCR plate, the following standards and controls were included: a calibrator sample, a negative control (DNAse and RNase free water) and a five-step 1:4 serial dilution of the calibrator DNA. A liquid handling robot (Freedom EVO by TECAN) was used to load all controls and all samples (1 ng) in random order and in triplicate onto 384 well qPCR plates. The robot mixed standards and samples with the qPCR master mix containing 5 μl of LightCycler 480 SYBR Green I Master (Roche) per well and the primers tel 1b (5’-CGG TTT GTT TGG GTT TGG GTT TGG GTT TGG GTT TGG GTT-3’) and tel 2b (5’-GGC TTG CCT TAC CCT TAC CCT TAC CCT TAC CCT TAC CCT-3’) (Epel et al., 2004) both at a concentration of 900 nmol or B2M primers (PrimerDesign, accession code NM_001009284) at a concentration of 300 nmol. This primer pair is designed to yield a 76 bp long product (Cawthon, 2002).

The following qPCR protocol was used on a LightCycler 480 (Roche): 15 min at 95°C (enzyme activation) followed by 50 cycles of 15 s at 95°C (denaturation), 30 s at 58°C (primer annealing), and 30 s at 72°C (signal acquisition). The melting curve was acquired as follows: 1 min at 95°C was followed by 30 s at 58°C and a continuous increase of 0.11°C/s to 95°C with continuous signal acquisition.

Amplification curves were baseline corrected using the software LinReg PCR (Ruijter et al., 2009), which was also used to calculate reaction-specific qPCR efficiencies for each plate (E_TEL_ and E_B2M_ for the telomere and the B2M reaction, respectively). The following formula was used for TL calculation (Pfaffl et al., 2002): 

(1) $$\mathrm{TL}=\frac{E_{\mathrm{TEL}}^{Cq_{\mathrm{TEL}(\mathrm{Calibrator})}-Cq_{\mathrm{TEL}\;(\mathrm{Sample})}}}{E_{B2M}^{Cq_{B2M(\mathrm{Calibrator})}-Cq_{B2M\;(\mathrm{Sample})}}}$$

where the Cq value describes the number of cycles of a qPCR that is required for an amplification curve to cross a set fluorescence threshold; the Cq values of the calibrator sample were Cq_TEL(Calibrator)_ and Cq_B2M(Calibrator)_ for the telomere and the B2M reaction, respectively; and Cq values of the individual samples were Cq_TEL(Sample)_ and Cq_B2M(Sample)_.

Samples had to pass the following qPCR quality control to be considered in the statistical analysis: the coefficient of variation calculated for the Cq values across triplicate had to be smaller than 5%, and individual amplicon efficiencies had to be within 5% of the reaction specific mean efficiency calculated using LinReg PCR.

Two telomere measurements were calculated: TL at birth (TLB), based on the first sample collected within 2 weeks after birth, and TL in first lactation (TLFL), based on the first sample collected within the milking period following the cow’s first calving. The records were log-transformed to ensure normality of the data distribution. All analyses and results reported henceforth are based on log-transformed TL measurements. 

### Genotyping

DNA was extracted from whole blood using the DNeasy Blood & Tissue kit (QIAGEN) as described in Seeker et al. (2018b). Animals used in the presented study had been previously genotyped under the following platforms:

- Illumina BovineSNP50 BeadChip (Illumina Inc., San Diego, CA)—246 animals
- GGP Bovine 50K (Neogen/GeneSeek)—161 animals
- GGP Bovine HD 150K (Neogen/GeneSeek)—295 animals

The genotypes obtained from the GGP chips 50K and 150K were subsequently imputed to the SNPs found on the 50K Illumina chip using the method of VanRaden et al. (2015) and the software FindHap.

### Factors Affecting Telomere Length and Genetic Parameter Estimation

A range of variables was extracted from the farm database to test their possible effect on TLB and TLFL (**Supplementary Table S1**). In addition, the qPCR well and plate were tested for impact on TL measurement.

A mixed linear model was fitted to the data, including the animal as random effect and the factors listed in **Supplementary Table S1** as fixed effects. The significance of the latter was determined using the P-value calculated for the conditional Wald’s F statistic. Selection of the effects for the best fitting model was done through an iterative backward elimination of effects with the highest probability value, with the random animal effect being constantly included. Animal genetic and feeding groups (feeding group of the dam for TLB), which were the main factors in the existing experimental design, were always retained in the model.

Once the fixed part of the model was decided, several random effects were tested. Firstly, a simple animal model was fitted including the significant fixed effects from the previous step and the animal random direct additive genetic effect, which was assumed to be normally distributed with parameters $N(0,\;\sigma_{A}^{2}A)$, where $\sigma_{A}^{2}$ is the additive genetic variance and ***A*** is a numerator relationship matrix based on animal pedigree. Heritability was estimated as the proportion of the total phenotypic variance explained by the direct additive genetic variance. Secondly, a random maternal genetic effect of the animal’s dam, which was assumed normally distributed with parameters $N(0,\;\sigma_{M}^{2}A)$, where $\sigma_{M}^{2}$ is the maternal genetic variance, was added to the model. Finally, a random maternal environment effect, which was assumed to be normally distributed as $N(0,\;\sigma_{\mathrm{me}}^{2}I)$, where $\sigma_{\mathrm{me}}^{2}$ is the variance explained by maternal environment and ***I*** the identity matrix, was also tested in a model with direct additive genetic effect. For both maternal genetic and maternal environments, the proportion of the total phenotypic variance explained by each term was calculated. The significance of each random effect was tested using the log-likelihood ratio test, with an effect deemed significant when twice the difference between the log-likelihood values of a model including and a model excluding the effect exceeded 3.84, which is the critical value of a chi-squared distribution at P<0.05 with 1 degree of freedom.

Subsequently, bivariate analyses between TLB and TLFL were conducted using the same models in order to derive estimates of the genetic, residual and phenotypic correlations between the two traits. Vector of the random effects was assumed to be MVN (0, ***V*** ⊗ ***A***), where ***V*** is the (co)variance matrix of the two traits. The null hypotheses were that the genetic correlation between TLB and TLFL is equal to unity and residual correlation is equal to zero. The hypotheses were tested using the log-likelihood ratio test, with the likelihoods compared to the ones obtained from the analyses where the correlations were fixed as defined by the null hypothesis.

All mixed linear models for statistical analyses were fitted to the data using the ASReml 4.0 package (Butler et al., 2017).

### Genome-Wide Association Studies

The SNP genotype data were subjected to quality control measures using PLINK v1.09 (Purcell et al., 2007): minor allele frequency >0.05, call rate >0.95, and Hardy–Weinberg equilibrium (P >10^−6^). After quality control, 44,160 SNP markers remained for further analysis.

Population stratification was investigated using a genomic relatedness matrix generated from all genotyped individuals. This matrix was converted to a distance matrix that was used to carry out classical multidimensional scaling analysis with the GenABEL package in R (Aulchenko et al., 2007), in order to obtain its principal components.

The GEMMA algorithm (Zhou and Stephens, 2014) was then used to perform GWAS with a standard univariate linear mixed model, which included the same fixed effects as described before, the first two principal components from the multidimensional scaling analysis to adjust for population structure, and the genomic relatedness matrix among individuals as a polygenic effect. After Bonferroni correction for multiple testing, significance thresholds were P ≤ 1.13 × 10^−6^ and P ≤ 2.26 × 10^−5^ for genome-wide significant (nominal P ≤ 0.05) and suggestive (namely, one false positive per genome scan) levels, respectively, corresponding to −log_10_(P) of 5.94 and 4.64. In addition, a search for SNPs significant at the chromosome-wide level (nominal P ≤ 0.05) was performed. The extent of linkage disequilibrium between significant SNPs located on the same chromosome was calculated using the r-square statistic of PLINK v1.09 (Purcell et al., 2007).

Individual markers found to be significant in GWAS were further examined with a mixed model that included the same fixed effects as before, an additional fixed effect of the corresponding SNP locus genotype and the random effect of the animal including pedigree-based relationships in a numerator relationship matrix. This analysis enabled the estimation of the genotypic effect from which additive and dominance genetic effects and the proportion of variance explained by each SNP locus were derived as follows: additive effect, a = (AA − BB)/2; dominance effect, d = AB − [(AA + BB)/2]; proportion of phenotypic variance due to SNP = [2pq (a + d(q − p))2]/VP, where AA, BB, and AB represent the predicted trait values for the corresponding genotype (AA=homozygous 1, BB=homozygous 2, and AB=heterozygous); p and q, the SNP allele frequencies; and VP, the total phenotypic variance of each trait derived in the genetic parameter estimation step. These analyses were performed using ASReml 4.0 software (Butler et al., 2017)

All genome-wide significant (post-Bonferroni correction) and suggestive significant SNPs identified in the genomic analysis for TLB and TLFL were mapped to the reference genome and annotated using the variant effect predictor (http://www.ensembl.org/Tools/VEP) tool within the Ensembl database and the Bos_taurus_UMD3.1 assembly. Moreover, genes located around the significant markers, in 0.5 Mb upstream and downstream windows, were annotated using the BioMart data-mining tool (http://www.ensembl.org/biomart/martview/) and the Bos_taurus_UMD3.1 assembly.

### Telomere Length and Animal Fitness

The associations of TLB and TLFL with future animal longevity, survival, health and reproductive traits were examined. These traits pertain to specific animal functions that vary between cows even when raised under similar conditions. For the purposes of the present study, these traits were collectively termed animal fitness traits. Details are presented in the **Supplementary Table S2**.

Phenotypic, genetic and residual correlation estimates between TL and animal fitness traits were obtained from bivariate analyses based on mixed models including the same effects for TL as described above and the effects shown in **Supplementary Tables S3**–**S5** for the different fitness traits. In a separate series of analyses, TL measurements were included as covariates in univariate analyses of fitness traits, to determine the phenotypic effect of the former on the latter. 

As animal fitness included traits with both continuous and binary values, two types of models were fitted: mixed linear models to continuous traits and generalized linear models with a logit link function to binary traits. For the latter, a threshold model was assumed, with results pertaining to the underlying liability scale. The significance of the TL measurement fitted as a covariate in continuous trait analyses was established from the P-value for the conditional Wald F statistic, and from a two-tailed t-test of the estimate for binary traits. The significance of correlations was based on the log-likelihood ratio test for continuous traits and a t-test for analyses where TL was fitted alongside a binary trait.

Due to the large number of biologically related traits used in the analyses, a significance threshold was corrected for multiple testing using sequentially rejective Holm–Bonferroni test (Holm, 1979). To establish the number of independent traits (and hypotheses) in the analyses, a principal component analysis was conducted on normalized data. This analysis was performed separately for the binary and continuous traits, classified based on the magnitude of the variance. The resulting number of clusters was seven and two for the continuous and binary traits, respectively, which amounted to the number of independent hypothesis tests considered per cluster.

## Results

### Factors Affecting Telomere Length Measurements

Of all factors tested (**Supplementary Table S1**), the qPCR well row and plate had a statistically significant (P < 0.05) effect on both TLB and TLFL. Lameness status of the dam during pregnancy was also found to significantly affect (P<0.05) TLB of her offspring, with calves of lame dams having shorter TLB than those of healthy dams. These fixed effects were fitted in the subsequent models of analysis together with the experimental design variables and genetic and feeding groups, to derive variance components and genetic parameters for the TL traits.

### Genetic Parameters of Telomere Length Traits

**Table 1** presents the estimates of variance components and genetic parameters for TLB and TLFL. Heritability estimates for TLB and TLFL were significantly (P < 0.05) greater than zero and moderate at 0.36 and 0.46, respectively. The maternal variance components were not significantly different from zero for any of the TL traits.

**Table 1:** Estimates of variance components and genetic parameters of telomere length at birth (TLB) and first lactation (TLFL) using different combinations of random effects (standard errors, in parentheses).

	TLB			TLFL		
	A	M	IdeM	A	M	IdeM
**V** **_P_**	0.020 (0.001) *	0.020 (0.001) *	0.020 (0.001) *	0.023 (0.002) *	0.023 (0.002) *	0.023 (0.002) *
**V** **_A_**	0.007 (0.002) *	0.007 (0.002) *	0.007 (0.002) *	0.011 (0.004) *	0.011 (0.004) *	0.011 (0.004) *
**V** **_M_**	–	0.000 (0.000)	0.000 (0.001)	–	0.000 (0.000)	0.000 (0.000)
**V** **_E_**	0.013 (0.002) *	0.013 (0.002) *	0.012 (0.002) *	0.013 (0.003) *	0.013 (0.003) *	0.013 (0.003) *
**h** **^2^**	0.36 (0.09) *	0.36 (0.09) *	0.35 (0.09) *	0.46 (0.15) *	0.46 (0.15) *	0.46 (0.15) *
**m** **^2^**	–	0.00 (0.00)	0.02 (0.06)		0.00 (0.00)	0.00 (0.00)

A, direct additive genetic effect model; M, as A with maternal genetic effect; IdeM, as A with maternal environmental effect; V_P_, total phenotypic variance; V_A_, additive genetic variance; V_M_, maternal variance, either genetic (model M) or environmental (model IdeM); V_E_, residual variance; h^2^, heritability; m^2^, proportion of total phenotypic variance explained by the maternal variance.

*Estimates greater than zero (P < 0.05).

**Figure 1:**
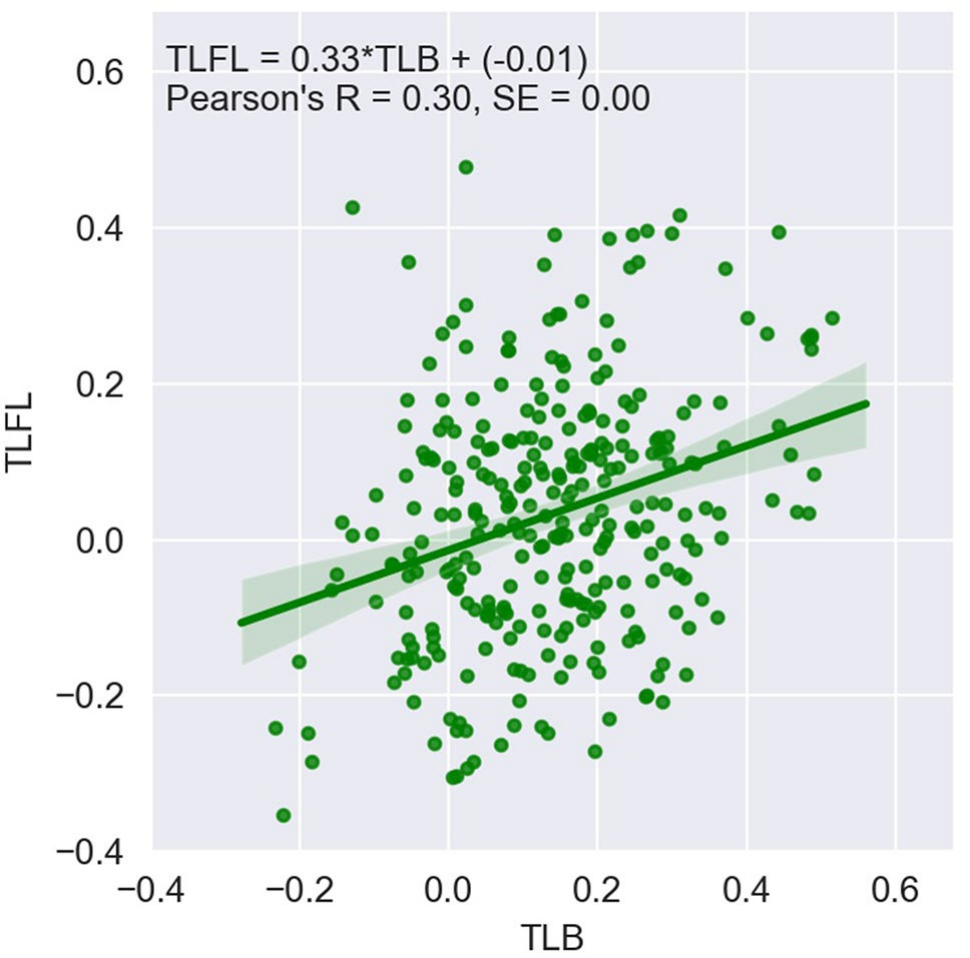
Telomere length at first lactation (TLFL) plotted against telomere length at birth (TLB); log-transformed values.

**Figure 1** shows TLFL plotted against TLB for 297 animals for which both records were available. TLB was significantly greater than TLFL (P < 0.05). The Pearson’s correlation between the phenotypes was 0.30 and significant (P < 0.05).

The bivariate analysis showed that TLB and TLFL are genetically highly correlated traits, with a genetic correlation of 0.78 (SE 0.14), which is not significantly different from unity. The phenotypic correlation was close to the correlation between raw values (**Figure 1**) at 0.35 (SE 0.06) and was significantly different from zero (P < 0.05). There was no detectable residual correlation between the two traits, with the estimate of −0.03 (SE 0.17) being not different from zero (P > 0.05).

### Genome-Wide Association Studies

Multidimensional scaling analysis revealed the presence of two principal components in the population corresponding to the two genetic groups, which were subsequently included in the GWAS model to account for population stratification.

**Figure 2:**
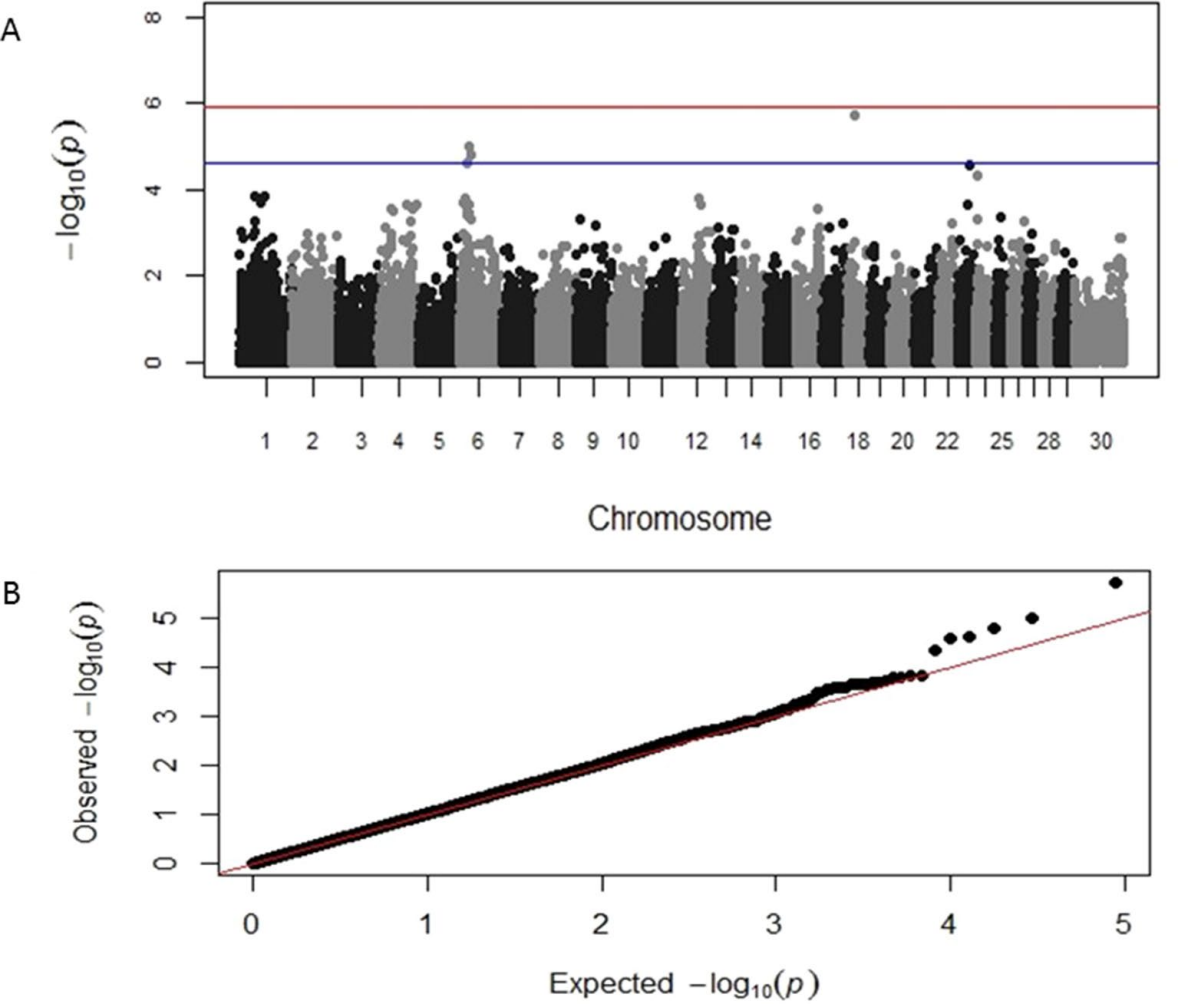
Manhattan and Q-Q plots displaying the genomic association results for telomere length at birth. **(A)** Genomic location is plotted against −log_10_(P). Red and blue lines, respectively, are thresholds for significance post-Bonferroni correction (P < 0.05) and for suggestive significance (accounting for one false positive per genome scan). **(B)** Observed P-values are plotted against the expected P-values.

**Figure 3:**
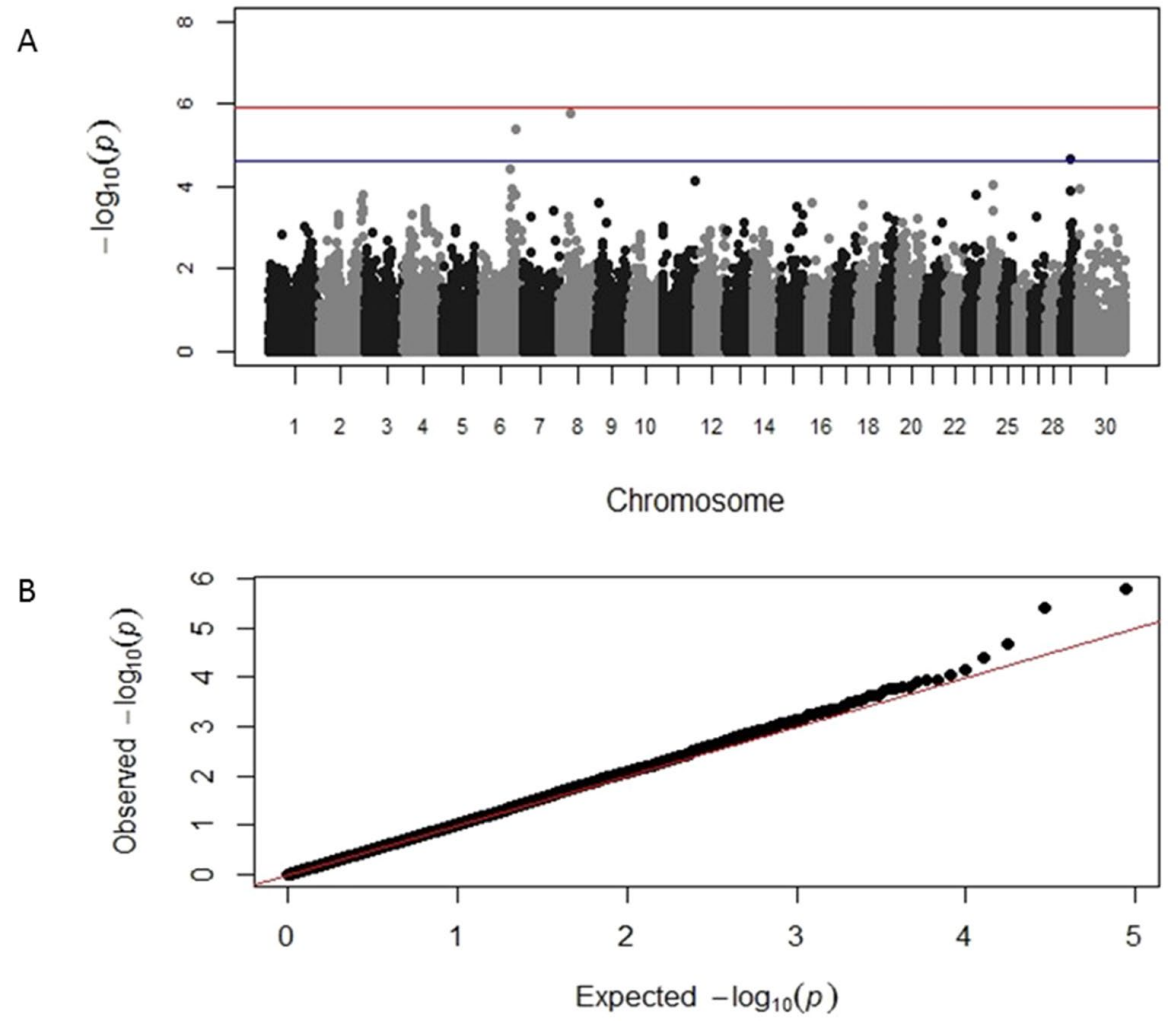
Manhattan and Q-Q plots displaying the genomic association results for telomere length at first lactation. **(A)** Genomic location is plotted against −log10(P). Red and blue lines, respectively, are thresholds for significance post-Bonferroni correction (P < 0.05) and for suggestive significance (accounting for one false positive per genome scan). **(B)** Observed P-values are plotted against the expected P-values.

GWAS results identified distinct genomic associations with the log-transformed TLB and TLFL measurements (**Table 2**). Specifically, three SNP markers were identified on chromosome 6, one on chromosome 18 and one on chromosome 23; all these markers had suggestive significant association with TLB. A chromosome-wide significant association of a SNP marker on chromosome 24 with TLB was also identified. In addition, three different SNP markers on chromosomes 6, 8 and 29, respectively, were identified having a suggestive significant association with TLFL (**Table 2**). The corresponding Manhattan and Q-Q plots for TLB and TLFL are displayed in **Figures 2** and **3**, respectively. The three markers identified on chromosome 6 for TLB were in close proximity and in high (>0.70) linkage disequilibrium with each other. These three SNPs were 70 MB from and not in linkage disequilibrium with the chromosome 6 SNP associated with TLFL.

**Table 2:** List of single nucleotide polymorphisms (SNPs) significantly associated with telomere length at birth (TLB) and first lactation (TLFL) in the studied population.

Trait	SNP	Chr (position)	P-value	Add (P-value)	Dom (P-value)	pVP
TLB	Hapmap52077-ss46526963	18 (24980984)	1.87E−06	0.06 (0.003)	0.015 (0.3)	0.03
	BTA-75768-no-rs*	6 (35147153)	1.02E−05	0.045 (0.00)	0.21 (0.15)	0.03
	Hapmap27329-BTC-055310*	6 (36655249)	1.61E−05	0.044 (0.003)	0.01 (0.02)	0.03
	Hapmap50091-BTA-75608*	6 (27433375)	2.43E−05	0.08 (0.02)	0.02 (0.64)	0.02
	ARS-BFGL-NGS-107030	23 (35503902)	2.69E−05	0.030 (0.05)	0.06 (0.003)	0.04
	Hapmap38789-BTA-86678	24 (9138102)	4.44E−05	0.021 (0.12)	0.04 (0.02)	0.00
TLFL	ARS-BFGL-NGS-53667	8 (35370804)	1.59E−06	0.073 (0.019)	0.011 (0.3)	0.04
	BTB-00279483	6 (106386383)	3.85E−06	0.075 (0.000)	0.002 (0.3)	0.10
	ARS-BFGL-NGS-43380	29 (28600601)	2.14E−05	0.077 (0.001)	0.03 (0.21)	0.11

Chr (position), chromosome number and position in bp; P-value, refers to the genome-wide association study; Add (P-value), refers to the additive effect; Dom (P-value), refers to the dominance effect; pVP, proportion of total phenotypic variance accounted for by the corresponding locus.

*Denotes SNPs in high linkage disequilibrium, essentially constituting proxies for one another.

All significant SNP markers that were identified in the GWAS were also found to have a significant effect on TLB and TLFL in the ensuing mixed model analyses based on the numerator relationship matrix. The additive and dominance genetic effects, and the proportion of the total phenotypic variance explained by each of these SNPs, are summarized in **Table 2**. A significant additive genetic effect was found in all loci with the exception of the one identified on chromosome 24; the latter, however, had a significant dominance effect on TLB. These significant SNP markers collectively explained approximately 10 and 25% of the phenotypic variance of TLB and TLFL, respectively.

### SNP and Candidate Region Annotation

Most significant SNPs identified in the GWAS were located in intronic, intergenic, and downstream gene regions. Only one of the SNPs (Hapmap52077-ss46526963) associated with TLB was localized in the 3’UTR region of the Nucleoporin 93 (*NUP93*) gene. All the above-mentioned variants were not coding variants and had a predicted modifier impact, meaning that prediction of impact may be difficult to make and can vary considerably (https://www.ensembl.org/info/genome/variation/prediction/predicted_data.html)

Most of the candidate regions for TLB and TLFL contained a small number of genes. In total, 12 protein-coding genes and two microRNAs were identified across the QTL regions for TLB, and nine protein-coding genes for TLFL (**Supplementary Table S6**).

### Associations Between Telomere Length and Animal Fitness

**Figure 4:**
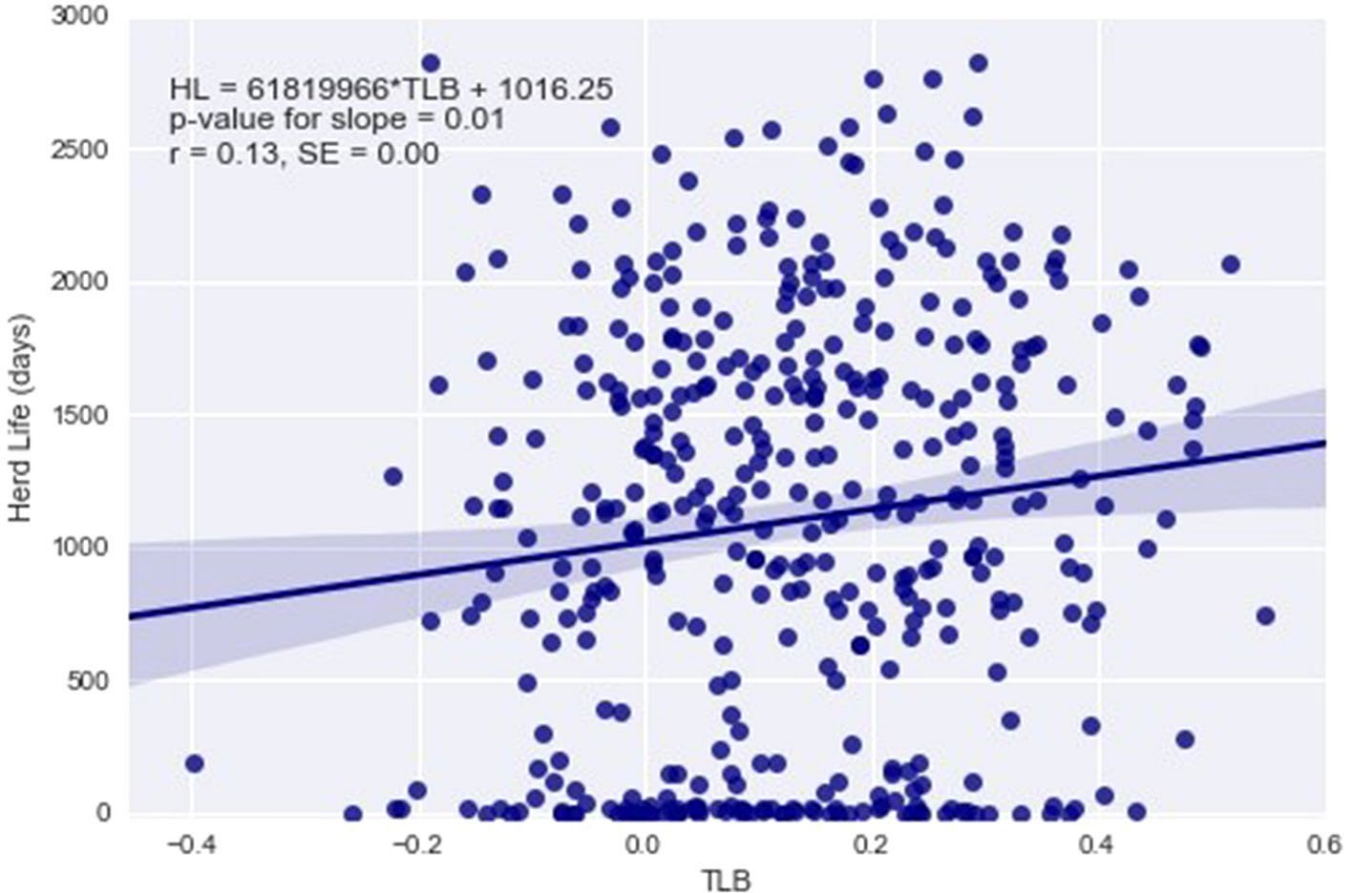
Regression of herd life (HL) defined as number of days from birth to death on telomere length at birth (TLB).

Statistically significant associations between TLB and animal fitness traits are summarized in **Table 3**. When fitted as a covariate, changes in TLB were found to significantly affect survival to 12 and 24 months of age and herd life, although only survival to 12 months maintained statistical significance after the Holm–Bonferroni correction (**Table 3**). Longer TLB were generally associated with higher chances of survival. This association was confirmed by the positive phenotypic correlations from the bivariate analyses between TLB and survival to ages of 24 and 48 months. For non-censored measures of lifespan, the significant regression of length of herd life on TLB (**Table 3**) is graphically illustrated in **Figure 4**, which implies a phenotypic correlation of 0.13 (SE 0.00) between the raw values of the two traits. When genetic co-variances between relatives were accounted for in the mixed model, significant genetic and residual correlations were found between TLB and survival to 48 months, at 0.76 (SE 0.23) and −0.21 (SE 0.07), respectively. This indicates that animals genetically predisposed for longer telomeres at birth are also genetically more likely to survive to 4 years of age. Interestingly, the residual correlation suggests that non-genetic factors that are favorable to one are unfavorable to the other trait. For health traits, the only significant estimate was the genetic and residual correlations between TLB and binary mastitis status (0.68 and −0.18, respectively), implying that animals with genes for higher TLB may be genetically more resistant to mastitis infection. As with survival, the residual correlation was weakly antagonistic. No significant associations between TLB and reproductive traits were derived from our analyses. Results of all TLB association analyses are presented in **Supplementary Tables S7**–**S9**. No significant associations of any kind were detected between TLFL and the studied animal traits.

**Table 3:** Regression coefficient (β) of telomere length at birth fitted as a covariate to fitness trait analyses, and corresponding correlation estimates from bivariate analyses (standard errors in brackets); only traits with at least one significant result included.

Trait	**β**	Bivariate analyses		
		R_A_	R_E_	R_P_
STM12	2.55 (0.85) **	0.52 (0.30)	−0.04 (0.04)	0.11 (0.07)
STM24	2.14 (0.75)*	0.87 (0.44)	−0.08 (0.04)*	0.12 (0.06)*
STM48	1.01 (0.99)	0.76 (0.23) **	−0.21 (0.07) **	0.13 (0.06)*
HL	531.4 (224.3)*	−0.40 (0.24)	0.15 (0.09)	0.01 (0.06)
MAST	−0.02 (0.80)	0.68 (0.28)*	−0.18 (0.05) **	0.07 (0.07)

STM12, 24, and 48: survival to 12, 24, and 28 months of age, respectively; HL, herd life measured in days between birth and death (cull); MAST, presence or absence of mastitis in all lactations

R_A_, genetic correlation; R_E_, residual correlation; R_P_, phenotypic correlation

* Statistically different from zero (P < 0.05) before but not after Holm–Bonferroni correction; ** Statistically different from zero (P < 0.05) after Holm–Bonferroni correction.

## Discussion

The present study examined the genetic background and genomic architecture of early-life TL measurements in dairy cows and their associations with future animal fitness traits related to survival, longevity, health and reproduction. The largest livestock population with TL measurements studied to date was used for this purpose. TLB and TLFL were found to be complex, polygenic, moderately heritable traits, and highly correlated to each other but associated with distinct genomic regions. Interestingly, TL measured within the first 2 weeks of life showed promising associations with survival, longevity and future health status of the cow, and could be used as an early-life biomarker for disease predisposition and lifespan in dairy cattle.

The statistically significant (P < 0.05) heritability estimates of 0.36 and 0.46 found for TLB and TLFL, respectively, confirmed earlier estimates in cattle (Seeker et al., 2018b) and fell within the wide range of estimates reported in other species, from 0.18 in collared flycatchers (Voillemot et al., 2012) to 0.82 in humans (Jeanclos et al., 2000). In contrast to studies performed in humans and wild species of birds and lizards, individual animals in the present study were kept in a standardized environment, with a detailed recording system in place monitoring their life events. The high heritability estimates obtained from human studies are notoriously affected by inflated estimates of genetic variance due to confounding with shared environment within families, which can be as high as or higher than the direct additive genetic component: for example, in a study of female di-zygotic twins, the estimates of the common environment and direct heritability were 0.49 and 0.36, respectively (Andrew et al., 2006). Such confounding is usually of little importance in dairy cattle, where calves are separated from their dams soon after birth. In the present study, TLB was on average longer than TL later in life (TLFL), which is in accordance to previous studies in other species (Slagboom et al., 1994;Bakaysa et al., 2007).

GWAS results shed more light on the genetic architecture of TL in dairy cows, highlighting specific genes that might underlie TL control. This is the first study, to our knowledge, that GWAS for TL traits were performed in a non-human mammal population. There are many previous GWAS of leukocyte TL in humans (Prescott et al., 2011; Codd et al., 2013; Pooley et al., 2013; Saxena et al., 2014; Delgado et al., 2018; Zeiger et al., 2018). However, specific GWAS of early-life TL are absent even from the human literature. The present study confirmed the findings of human studies showing that both TLB and TLFL are complex polygenic traits. The genomic associations identified here were distinct from those identified in the human studies. Moreover, distinct genomic associations and putative candidate genes were identified for the two TL measurements implying that the underlying molecular mechanism controlling the two traits is probably not identical in bovine. 

Specifically for TLB, the SNP marker on chromosome 18 was located in the 3’ UTR of the *NUD93* gene. The product encoded by *NUD93* is an important component of the nuclear pore complex in eukaryotic cells and a target of caspase cysteine proteases that play a central role in programmed cell death by apoptosis (Grandi et al., 1997). A recent study has reported that components of the nuclear pore complex play a key role in sub-telomeric gene silencing and, therefore, in TL (Van De Vosse et al., 2013). The SNPs on chromosome 6 associated with TLB were located in the intronic region of the coiled-coil serine rich protein 1 (*CCSER1*, alias *FAM190A*) gene. Deficiency of the coiled-coil serine rich protein 1 has been already associated with a cell division defect in humans and several human cancers (Patel et al., 2013). The SNP associated with TLB located on chromosome 23 is in close proximity with the *HDGFL1* gene. The hepatoma-derived growth factor protein of families in humans has been associated with cell proliferation after translocation to the nucleus (Kishima et al., 2002) and several forms of cancer (Chen et al., 2015; Shetty et al., 2016; Min et al., 2018). The only gene located close to the SNP with the most significant association with TLFL was *PTPRD*. The product of this gene, protein tyrosine phosphatase receptor type D, is an enzyme known to regulate a variety of cellular processes including cell growth, differentiation, mitotic cycle, and oncogenic transformation. The *PTPRD* gene has been also associated with different cancer forms in humans (Purdie et al., 2007; Veeriah et al., 2009; Zhao et al., 2015). Finally, the coiled-coil domain containing complexes play a critical role in genome organization and function (Poon and Mekhail, 2011), including the formation of heterochromatic domains within sub-telomeres, which are important for telomere function. Moreover, coiled-coil domain containing proteins are age-correlated DNA methylation markers in humans (Park et al., 2016). Interestingly, different members of this protein family (*CCDC15*, *CCDC102B*) were identified in close proximity with the SNP markers on chromosomes 29 and 24 associated with TLB and TLFL in the present study, respectively. All the above discussed genes are putative good candidates for TLB and TLFL in dairy cattle; however, further studies on independent datasets are needed to validate these results and identify the causative mutations.

Our results may also be informative for the telomere biology of humans and other species. Alongside the additive genetic components of variance, we have explored the maternal effects on TL. Preliminary results within the present study using a subset of the data (n = 308) had detected a significant maternal permanent environment effect, explaining 33% of the total variance, which is of similar magnitude to the direct heritability estimate (h^2^ = 0.36) (Ilska et al., 2017). However, as TLB measurements of new individuals were added to the data, the estimate of the permanent environment became non-significant. There is no obvious explanation for this. The inconsistency of the estimates for maternal effects is common in studies of TL. While the concept of paternal inheritance has gained support over decades (Njajou et al., 2007; Nordfjäll et al., 2005; Nordfjäll et al., 2009), recently, an epigenetic mode of inheritance was proposed (De Meyer et al., 2014) after a large meta-analysis had detected a stronger maternal inheritance of TL in humans (Broer et al., 2013).

In addition to the maternal effects, we also examined lactation-dependent dam effects and covariates. As the TLB was measured very early in life, it was expected that *in utero* conditions would have the highest impact on the trait in the calf. Previous studies in other species have indicated that maternal diet (Jennings et al., 1999), stress levels (Entringer et al., 2013; Marchetto et al., 2016) and hormone levels (Tissier et al., 2014) during pregnancy may affect TL in offspring. In the present study, the effect of the dam’s feeding group on her offspring’s TLB was tested and found to be non-significantly different from zero (P > 0.05). This can be explained by the fact that both diets administered to the cows of the study were species-appropriate and non-limiting of the cow’s basic biological functions, as compared to distinctly “unhealthy” diets examined in human studies, where increased consumption of products such as processed meats, cereals and sweetened beverages has showed an association with shorter TL (Rafie et al., 2016). On the other hand, the significant effect reported here of the lameness status of the dam during pregnancy on her offspring’s TLB supports previous findings on the association between *intra-uterine* stress, hormone exposure, and TL in offspring. Lameness is a major cause of chronic stress in bovine and has been shown to affect the hormonal cascade, particularly levels of cortisol (Gellrich et al., 2015) and estradiol (Dobson et al., 2008). Cortisol is a typical stress hormone, which is involved in the hypothalamic–pituitary–adrenal axis, has been shown to elevate the oxidative stress, and affect levels of telomerase and, subsequently, TL (Choi et al., 2008; Tissier et al., 2014). Estradiol, specifically 17 β-estradiol, activates the catalytic subunit of telomerase, which is the enzyme responsible for telomere maintenance (Kyo et al., 1999).

The significant residual and suggestive genetic correlations between TLB and clinical mastitis status of the cow provide a promising result. While associations between TL and inflammation and infection, which are both involved in clinical mastitis, have been previously reported in humans (Cawthon et al., 2003), the association of an early-life TL measurement with an inflammatory disease in an adult has not been studied before. Furthermore, previous understanding of the association between telomeres and immunity was based on the increased rates of cell division in diseased states, leading to faster telomere attrition (De Martinis et al., 2006). Here, we present the associations between an early-life, single-point measure (TLB), and an inflammatory disease later in life. Of particular interest is the positive genetic correlation between the two traits: a confirmed genetic correlation could markedly facilitate genetic selection for mastitis resistance by using the earlier measured and more heritable TLB; selection for longer TLB could positively affect udder health.

Moreover, the high genetic correlation found between TLB and survival to 48 months (0.76, P < 0.05) could accelerate genetic selection for enhanced cattle longevity. While we also detected significant and positive phenotypic correlations between TLB and survival to 12, 24, and 36 months, more research is needed to further dissect the associations of TB with survival to later ages, which can determine the profitability of the cow and sustainability of the farm. In general, long TL was associated with enhanced longevity and survival at either the genetic or phenotypic level. Previously published associations of TL with longevity are inconclusive and depend on the species and population studied, the animal age at TL measurement, and the definition of longevity. The associations between longevity and TL in dairy cows are likely to be different to those detected in humans and wild animals, as the longevity of a dairy cow does not represent her biological limits but instead depends on her productivity and reproduction—both of which carry high oxidative stress (Metcalfe and Alonso-Alvarez, 2010). Brown et al. (2012) detected a significant but weak phenotypic association between TL and cow survival in the following year on a sample of 285 cows, of which 58 were culled in the period of study. Seeker et al. (2018a) found that bovine TL at 1 year of age is phenotypically correlated with lifespan. Compared to both these studies, by increasing the number of records, we were able to show here that TLB is a useful candidate biomarker of early- and mature-life survival.

The present study focused on specific early life TL measurements. Future studies should examine the genetic background of the rate of telomere shortening over time, also known as attrition rate, and the association with animal performance and fitness in later life.

In conclusion, the present study examined the genetic and genomic architecture of bovine TL at birth and in first lactation and found them to be moderately heritable, complex polygenic and highly correlated traits. A comprehensive examination of the associations between TL and animal fitness traits expressed at both early and mature age provided evidence of an association of TLB with survival, longevity and mastitis resistance. These findings may be used to inform breeding programs and strengthen the interest for further studies to fully understand the reported associations and the underlying mechanism of TL changes over time.

## Data Availability Statement

The datasets for this manuscript are not publicly available because the animals, and associated records and sampling, are part of a long term (50 years) genetic selection experiment supported by a range of funders including commercial and confidential in nature. As such the data in their entirety are not available to upload to a public database. Instead, we have established a publically facing interface (https://www.egenes.co.uk/langhillUP/) where all requests to access the datasets can be made for research purposes. All reasonable research-focused requests are normally accepted.

## Ethics Statement

The animal study was reviewed and approved by Blood sample collection was conducted in accordance with UK Home Office regulations (PPL No: 60/4278 Dairy Systems, Environment and Nutrition) and procedures were approved by the SRUC Animal Experimentation Committee.

## Author Contributions

JI-W performed the genetic parameter and trait association analyses. AP performed the genomic and post-genomic analysis. LS, RW, SU, JF and DN performed the telomere length measurements. JI-W collated the blood sample catalogue, curated the samples, and constructed the database of phenotypic and telomere length records for the purpose of the experiment. JI-W, GB and AP collated and edited the genotyping data. All authors met routinely during the project and contributed to the analysis design and interpretation of results. JI-W and AP wrote the manuscript with input from GB. All other co-authors provided manuscript editing and feedback. All authors read and approved the final manuscript. GB, MC, DN, BW and AP conceived and designed the genetic and genomic studies and secured funding.

## Funding

The authors gratefully acknowledge funding from the Biotechnology and Biological Sciences Research Council (BB/L007312/1 and BBS/E/D/30002276; http://www.bbsrc.ac.uk/), the Rural & Environment Science & Analytical Services Division of the Scottish Government, and Scotland’s Rural College (http://www.sruc.ac.uk/).

## Conflict of Interest

The authors declare that the research was conducted in the absence of any commercial or financial relationships that could be construed as a potential conflict of interest.
