# Supplementary material for: The Genetic Architecture of Bovine Telomere Length in Early Life and Association With Animal Fitness
Source: Front Genet. 2019 Oct 25;10:1048. doi: 10.3389/fgene.2019.01048 (PMC6843005; doi:10.3389/fgene.2019.01048)
Supplement: Supplementary file 1 [file DataSheet_1.docx]

Supplementary Material

Supplementary Table S1: Description of effects fitted to the analysis of telomere length record at birth (TLB) and during first lactation (TLFL).

| Effect | Description | TLB | TLFL |
| --- | --- | --- | --- |
| GenGroup | Genetic group: selected (S) and controls (C) | ✓ | ✓ |
| FeedGroup | Feeding group: 1 – low forage, 2 – high forage | ✓ (dam) | ✓ |
| LACT_BORN | Lactation born – calves born in lactations >5 grouped into one category | ✓ | ✓ |
| CALVING_EASE | Calving ease - all forms of assisted calving grouped into one category, resulting in binary variable | ✓ | ✓ |
| HOLST | Percentage Holstein |  | ✓ |
| BIRTH_WGT | Birth weight of the calf (kg) | ✓ | ✓ |
| YSOB | Year/season of birth – two seasons per year, October- March and April-September | ✓ | ✓ |
| YSOC | Year/season of calving – two seasons per year, October-March and April-September |  | ✓ |
| ageAtFirstAI | Age (in days) at first Artificial Insemination |  | ✓ |
| ageAtCalf | Age at calving | ✓ (dam, years) | ✓ (days) |
| AGE_M | Age (in months) at blood sample collection |  | ✓ |
| GESTATION | Gestation length (days) | ✓ (dam) | ✓ |
| CALVING_WGT | Calving body weight (kg) | ✓ (dam) | ✓ |
| CALVING_BCS | Body condition score at calving (1-5 scale) | ✓ (dam) | ✓ |
| FERT | 0 – dam underwent fertility treatment during pregnancy, 1 – no treatment | ✓ (dam) | ✓ |
| LAME | 0 – dam suffered lameness during pregnancy, 1 – no lameness | ✓ (dam) | ✓ |
| MAST | 0 – dam suffered mastitis during pregnancy, 1 – no mastitis | ✓ (dam) | ✓ |
| PLATE | qPCR plate | ✓ | ✓ |
| WellRow | qPCR well row | ✓ | ✓ |
| damMY | Total milk production of the dam in lactation (limited to 305 days) preceding birth of the calf | ✓ (dam) |  |
| damSCC | Somatic cell count of the dam in the preceding lactation | ✓ (dam) |  |
| damEPISODES | Total number of fertility treatments, lameness and mastitis in the preceding lactation | ✓ (dam) |  |
| YSOB.LAME | Interaction between year/season of birth and lameness status of the dam | ✓ |  |
| YSOB.damFEED | Interaction between year/season of birth and feeding group of the dam | ✓ |  |

Supplementary Table S2: Animal fitness traits.

| Trait Group | Abbreviation | Description |
| --- | --- | --- |
| Longevity and survival | HL | Herd life measured in days between birth and death (cull) |
|  | HLfunc | Herd life measured in days between birth and death (cull) corrected for milk production |
|  | PL | Productive life measured in days between first calving and death (cull) |
|  | PLfunc | Productive life measured in days between first calving and death (cull) corrected for milk production |
|  | STM | Censored survival to a certain age in months (M), ranging from 12 to 72 in 12-month intervals |
| Health | LAME | 1. Number of distinct episodes of lameness (cases occurring within 28 days were treated as the same episode) 2. Presence or absence of condition   both recorded separately in first lactation and across all lactations |
|  | MAST | 1. Number of distinct episodes of mastitis (cases occurring within 7 days were treated as the same episode) 2. Presence or absence of condition   both recorded separately in first lactation and across all lactations |
|  | FERT | 1. Number of distinct episodes of fertility disorders (cases occurring within 21 days were treated as the same episode) 2. Presence or absence of condition   both recorded separately in first lactation and across all lactations |
| Reproductive | DAFS | Age in days at first service (insemination) |
|  | L0INS | Number of inseminations per conception before 1^st^ calving |
|  | L1INS | Number of inseminations per conception after 1^st^ and before 2^nd^ calving |
|  | L0CR | Conception rate recorded as binary success at the first insemination |
|  | L1CR | Conception rate recorded as binary success at the first insemination after first calving |
|  | CI | Calving interval measured as days between 1^st^ and 2^nd^ calvings |
|  | DYST | Dystocia at 1^st^ calving recoded as binary trait reflecting easy and difficult calvings |
|  | DAFC | Age in days at first calving |

Supplementary Table S3: Fixed effects considered in the analyses of animal longevity and survival traits, and corresponding heritability (h^2^) estimates. For survival traits marked with a *, the heritability reported was obtained from binomial analysis and represents heritability on the underlying liability scale; model effect and trait definitions are in Supplementary Tables S1 and S2, respectively.

| Animal trait | Model effects | h^2^ (SE) |
| --- | --- | --- |
| HL | GenGroup, YSOB, HOLST, BIRTH_WGT, FeedGroup, | 0.18 (0.03) |
| PL | GenGroup, L1_YS, FeedGroup, | 0.18 (0.06) |
| HLfunc | GenGroup, YSOB, HOLST, BIRTH_WGT, FeedGroup | 0.16 (0.06) |
| PLfunc | GenGroup, L1_YS, FeedGroup | 0.19 (0.06) |
| STM12* | GenGroup, YSOB, HOLST, BIRTH_WGT | 0.27 (0.04) |
| STM24* | GenGroup, YSOB, HOLST, BIRTH_WGT | 0.22 (0.03) |
| STM36* | GenGroup, YSOB, HOLST, BIRTH_WGT | 0.18 (0.03) |
| STM48* | GenGroup, YSOB, HOLST | 0.15 (0.02) |
| STM60* | GenGroup, YSOB, HOLST | 0.12 (0.02) |
| STM72* | GenGroup, YSOB | 0.11 (0.02) |

Supplementary Table S4: Fixed effects considered in the analyses of animal health traits and corresponding heritability (h^2^) estimates. All traits were recorded either as the number of distinct episodes or as a binary variable (presence or absence of the condition); for the latter, the heritability reported was obtained from binomial analysis and represents heritability on the underlying liability scale; model effect and trait definitions are in Supplementary Tables S1 and S2, respectively.

| Animal trait | Time period | Model effects | h^2^ (SE) | Binary h^2^ (SE) |
| --- | --- | --- | --- | --- |
| FERT | 1^st^ lactation | GenGroup, FeedGroup, YSOC | 0.08 (0.04) | 0.10 (0.05) |
|  | All lactations | GenGroup, FeedGroup, YSOC | 0.10 (0.04) | 0.12 (0.07) |
| LAME | 1^st^ lactation | GenGroup, FeedGroup, YSOC | 0.08 (0.03) | 0.08 (0.04) |
|  | All lactations | GenGroup, FeedGroup, YSOC | 0.11 (0.04) | 0.06 (0.04) |
| MAST | 1^st^ lactation | GenGroup, FeedGroup, YSOC | 0.03 (0.03) | 0.08 (0.05) |
|  | All lactations | GenGroup, FeedGroup, YSOC | 0.08 (0.03) | 0.10 (0.03) |

Supplementary Table S5: Fixed effects considered in the analyses of animal reproductive traits, and corresponding heritability (h^2^) estimates. For L0CR, L1CR and DYST the heritability reported was obtained from binomial analysis and represents heritability on the underlying liability scale; model effect and trait definitions are in Supplementary Tables S1 and S2, respectively.

| Animal trait | Model effects | h^2^ (SE) |
| --- | --- | --- |
| DAFS | GenGroup, BIRTH_WGT, YSOB | 0.06 (0.02) |
| L0INS | GenGroup, YSOB | 0.04 (0.03) |
| L0CR | GenGroup, YSOB | 0.01 (0.02) |
| CI | GenGroup, FeedGroup, L1_YS | 0.09 (0.04) |
| DYST | GenGroup, FeedGroup, L1_YS | 0.09 (0.03) |
| DAFC | GenGroup, HOLST, FeedGroup, L1_YS | 0.12 (0.04) |
| L1INS | GenGroup, FeedGroup, L1_YS | 0.09 (0.04) |
| L1CR | GenGroup, FeedGroup, L1_YS | 0.01 (0.04) |

| **Supplementary Table S6.** Genes located in the candidate genomic regions identified for telomere length at birth (TLB) and first lactation (TLFL) in the studied population. | | | | | | |
| --- | --- | --- | --- | --- | --- | --- |
|  |  |  |  |  |  |  |
| **TLB** |  |  |  |  |  |  |
| **Gene stable ID** | **Transcript stable ID** | **Gene description** | **Chromosome number** | **Gene start (bp)** | **Gene end (bp)** | **Gene name** |
| ENSBTAG00000019808 | ENSBTAT00000026390 | coiled-coil serine rich protein 1 | 6 | 35100701 | 35938394 | CCSER1 |
| ENSBTAG00000010285 | ENSBTAT00000047683 | multimerin 1 | 6 | 36087557 | 36199876 | MMRN1 |
| ENSBTAG00000024957 | ENSBTAT00000021300 | synuclein alpha | 6 | 36285494 | 36432426 | SNCA |
| ENSBTAG00000045966 | ENSBTAT00000064473 | GPRIN family member 3 | 6 | 36989139 | 36991481 | GPRIN3 |
| ENSBTAG00000006611 | ENSBTAT00000008683 | nucleoporin 93 | 18 | 24879123 | 24981115 | NUP93 |
| ENSBTAG00000029938 | ENSBTAT00000042317 | bta-mir-138-2 | 18 | 24992696 | 24992779 | bta-mir-138-2 |
| ENSBTAG00000015130 | ENSBTAT00000020131 | HDGF like 1 | 23 | 35437310 | 35439298 | HDGFL1 |
| ENSBTAG00000044561 | ENSBTAT00000061994 | bta-mir-2284c | 23 | 35647120 | 35647199 | bta-mir-2284c |
| ENSBTAG00000042755 | ENSBTAT00000059747 |  | 23 | 35722114 | 35722222 | RF00026 |
| ENSBTAG00000046957 | ENSBTAT00000064953 | docking protein 6 | 24 | 7692283 | 8100346 | DOK6 |
| ENSBTAG00000036666 | ENSBTAT00000051166 |  | 24 | 8343335 | 8343443 | RF00001 |
| ENSBTAG00000006726 | ENSBTAT00000008842 | coiled-coil domain containing 102B | 24 | 8587478 | 8645504 | CCDC102B |
| ENSBTAG00000007567 | ENSBTAT00000009957 | thioredoxin related transmembrane protein 3 | 24 | 8748241 | 8785474 | TMX3 |
| ENSBTAG00000037907 | ENSBTAT00000056960 | dermatan sulfate epimerase-like | 24 | 9741024 | 9744662 | DSEL |
|  |  |  |  |  |  |  |
| **TLFL** |  |  |  |  |  |  |
| **Gene stable ID** | **Transcript stable ID** | **Gene description** | **Chromosome number** | **Gene start (bp)** | **Gene end (bp)** | **Gene name** |
| ENSBTAG00000010178 | ENSBTAT00000013433 | protein tyrosine phosphatase, receptor type D | 8 | 36575709 | 36797407 | PTPRD |
| ENSBTAG00000011163 | ENSBTAT00000014829 | cytokine like 1 | 6 | 105926864 | 105931259 | CYTL1 |
| ENSBTAG00000010875 | ENSBTAT00000014447 | msh homeobox 1 | 6 | 106061463 | 106065759 | MSX1 |
| ENSBTAG00000005712 | ENSBTAT00000007503 | syntaxin 18 | 6 | 106365304 | 106482788 | STX18 |
| ENSBTAG00000005711 | ENSBTAT00000007502 | neuronal vesicle trafficking associated 1 | 6 | 106483716 | 107356158 | NSG1 |
| ENSBTAG00000004893 | ENSBTAT00000044120 | acyl-CoA oxidase 3, pristanoyl | 6 | 106492148 | 106531843 | ACOX3 |
| ENSBTAG00000004797 | ENSBTAT00000006298 | tRNA methyltransferase 44 homolog | 6 | 106552949 | 106577901 | TRMT44 |
| ENSBTAG00000014803 | ENSBTAT00000019695 | carboxypeptidase Z | 6 | 106626199 | 106650047 | CPZ |
| ENSBTAG00000031197 | ENSBTAT00000044175 | H6 family homeobox 1 | 6 | 106868232 | 106872198 | HMX1 |
| ENSBTAG00000031197 | ENSBTAT00000056515 | H6 family homeobox 1 | 6 | 106868232 | 106872198 | HMX1 |

**Supplementary Table S7:** Regression coefficient (β) of TLB fitted as a covariate to animal longevity and survival trait analyses, and corresponding correlation estimates from bivariate analyses (standard errors in brackets); trait definitions are in Supplementary Table S2.

| Animal trait | β | Bivariate analyses | | |
| --- | --- | --- | --- | --- |
|  |  | **R_A_** | **R_E_** | **R_P_** |
| HL | **531.4 (224.3)*** | -0.40 (0.24) | 0.15 (0.09) | 0.01 (0.06) |
| HLfunc | 156.0 (179.5) | -0.11 (0.26) | 0.07 (0.09) | 0.02 (0.06) |
| PL | 97.01 (178.8) | 0.05 (0.28) | 0.04 (0.12) | 0.04 (0.07) |
| PLfunc | 2.06 (202.6) | 0.24 (0.27) | -0.06 (0.12) | 0.02 (0.07) |
| STM12 | **2.55 (0.85) **** | 0.52 (0.30) | -0.04 (0.04) | 0.11 (0.07) |
| STM24 | **2.14 (0.75)*** | 0.87 (0.44) | **-0.08 (0.04)*** | **0.12 (0.06)*** |
| STM36 | -1.18 (1.28) | -0.06 (0.26) | 0.08 (0.04) | 0.05 (0.04) |
| STM48 | 1.01 (0.99) | **0.76 (0.23) **** | **-0.21 (0.07) **** | **0.13 (0.06)*** |
| STM60 | 1.06 (0.91) | 0.26 (0.33) | 0.00 (0.05) | 0.04 (0.05) |
| STM72 | 0.01 (1.2) | -0.02 (0.38) | -0.08 (0.06) | -0.06 (0.08) |
| R_A_: genetic correlation; R_E_: residual correlation; R_P_: phenotypic correlation  *statistically different from zero (P<0.05) before but not after Holm-Bonferroni correction  ** statistically different from zero after Holm-Bonferroni correction | | | | |

Supplementary Table S8: Regression coefficient (β) of TLB fitted as a covariate to animal health trait analyses, and corresponding correlation estimates from bivariate analyses (standard errors in brackets); trait definitions are in Supplementary Table S2.

| Animal trait | Time period | β | Bivariate analyses | | |
| --- | --- | --- | --- | --- | --- |
|  |  |  | **R_A_** | **R_E_** | **R_P_** |
| FERT  presence/absence | 1^st^ lactation | NE | -0.43 (0.41) | **0.11 (0.05)*** | 0.00 (0.06) |
|  | All lactations | NE | NE | NE | NE |
| LAME  presence/absence | 1^st^ lactation | 0.26 (0.74) | 0.20 (0.47) | -0.01 (0.05) | 0.02 (0.06) |
|  | All lactation | 0.32 (0.82) | NE | NE | NE |
| MAST  presence/absence | 1^st^ lactation | 0.63 (1.08) | NE | NE | NE |
|  | All lactations | -0.02 (0.8) | **0.68 (0.28)*** | **-0.18 (0.05) **** | 0.07 (0.07) |
| FERT  no. episodes | 1^st^ lactation | NE | 1.20 (2.25) | -0.16 (0.09) | -0.03 (0.05) |
|  | All lactations | NE | NE | NE | NE |
| LAME  no. episodes | 1^st^ lactation | -0.25 (0.31) | -0.09 (0.27) | -0.02 (0.07) | -0.03 (0.05) |
|  | All lactations | -0.61 (0.70) | -0.11 (0.27) | -0.02 (0.08) | -0.04 (0.05) |
| MAST  no. episodes | 1^st^ lactation | 0.03 (0.14) | -0.66 (0.42) | 0.05 (0.07) | -0.03 (0.05) |
|  | All lactations | 0.08 (0.56) | -0.28 (0.28) | 0.03 (0.07) | -0.03 (0.05) |
| NE: Non-estimable  R_A_: genetic correlation; R_E_: residual correlation; R_P_: phenotypic correlation  *statistically different from zero (P<0.05) before but not after Holm-Bonferroni correction  ** statistically different from zero after Holm-Bonferroni correction | | | | | |

Supplementary Table S9: Regression coefficient (β) of TLB fitted as a covariate to animal reproductive trait analyses, and corresponding correlation estimates from bivariate analyses (standard errors in brackets); trait definitions are in Supplementary Table S2.

| Animal trait | β | Bivariate analyses | | |
| --- | --- | --- | --- | --- |
|  |  | **R_A_** | **R_E_** | **R_P_** |
| DAFS | 5.62 (14.31) | NE | NE | NE |
| L0INS | 0.14 (0.46) | 0.43 (0.36) | -0.06 (0.06) | 0.00 (0.04) |
| L0CR | 0.02 (0.67) | 0.22 (0.55) | -0.03 (0.05) | 0.00 (0.05) |
| DYST | 0.65 (0.76) | 0.04 (0.34) | 0.04 (0.05) | 0.04 (0.06) |
| DAFC | 8.68 (19.44) | 0.55 (0.29) | -0.12 (0.08) | 0.01 (0.05) |
| L1INS | -0.8 (0.86) | -0.50 (0.31) | 0.07 (0.07) | -0.03 (0.05) |
| L1CR | 0.08 (0.94) | -0.19 (0.46) | 0.02 (0.07) | -0.02 (0.07) |
| CI | -29.59 (26.89) | -0.38 (0.32) | 0.02 (0.08) | -0.04 (0.05) |
| R_A_: genetic correlation; R_E_: residual correlation; R_P_: phenotypic correlation | | | | |
